# Reinforcement of integrin-mediated T-Lymphocyte adhesion by TNF-induced Inside-out Signaling

**DOI:** 10.1038/srep30452

**Published:** 2016-07-28

**Authors:** Qian Li, Steven Huth, Dieter Adam, Christine Selhuber-Unkel

**Affiliations:** 1 Institute for Materials Science, Biocompatible Nanomaterials, Christian-Albrechts-Universität zu Kiel, Kaiserstr. 2, D-24143 Kiel, Germany; 2 Institute of Immunology, Christian-Albrechts-Universität zu Kiel, Michaelisstr. 5, D-24105 Kiel, Germany

## Abstract

Integrin-mediated leukocyte adhesion to endothelial cells is a crucial step in immunity against pathogens. Whereas the outside-in signaling pathway in response to the pro-inflammatory cytokine tumour necrosis factor (TNF) has already been studied in detail, little knowledge exists about a supposed TNF-mediated inside-out signaling pathway. In contrast to the outside-in signaling pathway, which relies on the TNF-induced upregulation of surface molecules on endothelium, inside-out signaling should also be present in an endothelium-free environment. Using single-cell force spectroscopy, we show here that stimulating Jurkat cells with TNF significantly reinforces their adhesion to fibronectin in a biomimetic *in vitro* assay for cell-surface contact times of about 1.5 seconds, whereas for larger contact times the effect disappears. Analysis of single-molecule ruptures further demonstrates that TNF strengthens sub-cellular single rupture events at short cell-surface contact times. Hence, our results provide quantitative evidence for the significant impact of TNF-induced inside-out signaling in the T-lymphocyte initial adhesion machinery.

Immunity against pathogens critically depends on the ability of leukocytes to rapidly transmigrate from the bloodstream into inflamed tissue and to the site of infection. Diapedesis occurs via a series of steps that require the integrin-mediated adhesion of leukocytes to endothelial cells of the blood vessel^1^. In detail, leukocytes are initially captured from the bloodstream through interactions that are mediated by L-selectin^2^ before they slowly start to roll on top of the endothelium, a process mediated by E-selectin, P-selectin, ICAM-1, and VCAM-1. Adhesion is mainly mediated by integrins on the surface of leukocytes, e.g. α_L_β_2_ on neutrophils and α_4_β_1_ on T cells and ICAM-1, and VCAM-1 on endothelial cells^3^. Hence, integrins are key players in mediating leukocyte adhesion to the endothelium of blood vessels.

Integrins naturally adopt a low-affinity, inactive conformation that needs to be converted to an active, high-affinity conformation for efficient cell adhesion. This conversion can either occur by binding to external ligands (outside-in signaling) or by interaction of the cytoplasmic domains of integrin chains with intracellular signaling cascades triggered by foreign antigens, chemokines or proinflammatory cytokines (inside-out signaling)^4^. As one of the most potent pro-inflammatory cytokines, tumour necrosis factor (TNF) mediates leukocyte adhesion to the endothelium by upregulating integrin ligands (e.g. ICAM-1, VCAM-1) on the surface of endothelial cells^5^. In addition to this well-established function of TNF as a trigger of integrin outside-in signaling, a recent study on neutrophils has implicated TNF in the activation of integrins by eliciting inside-out signaling^6^, supporting the notion that TNF can enhance the inflammatory recruitment of effector cells not only by activating the endothelium, but also by directly enhancing the adhesive properties of leukocytes themselves. In addition to neutrophils as important effectors of innate immunity, T cells represent another leukocyte population that is crucial for all adaptive immune responses. While the inside-out-mediated activation of integrins in T cells by chemokines or by the T cell receptor is well established^7^, a possible role of TNF in this process is much less explored. Alon *et al*. have reported that TNF can bind to the integrin ligand fibronectin and, as part of this complex, augment the β_1_ integrin-mediated adhesiveness of activated CD4-positive T cells^8^. However, the study did not address to what extent TNF-dependent inside-out or fibronectin-mediated outside-in signaling contributes to this process. Nevertheless, it is clear that combinatorial signals by inflammatory cytokines such as TNF and chemokines mediate the interaction of T cells and leukocytes with the extracellular matrix^9^. We have recently demonstrated that TNF can induce a signaling pathway in T cells that leads to activation of the lipase neutral sphingomyelinase 2 (nSMase2) and involves the proteins FAN, RACK1 and EED as transducing components^10^. Interestingly, loss of FAN causes a defective recruitment of leukocytes to sites of infection^11^, RACK1 and EED have been described as direct interaction partners of the cytoplasmic domains of integrins^12^,^13^,^14^, and nSMase2 has been implicated in the adhesion, migration and confluence-dependent contact inhibition of cells^15^. In summary, these findings led us to speculate that TNF may control the inflammatory recruitment and the binding of T cells to the endothelium by directly impacting on the adhesiveness of T cell integrins via inside-out signaling, in addition to its well-established effects on the outside-in signaling via endothelial cells.

Single-cell force spectroscopy (SCFS) has evolved in recent years as an excellent quantitative method to characterize cell-cell and cell-surface adhesion^16^,^17^. Particularly because of its potential to characterize cellular adhesion events from the single molecule level to the level of molecular clusters and whole cells, it is able to quantify a large number of parameters that are relevant to cell adhesion^18^,^19^,^20^,^21^. Using SCFS, the TNF-mediated outside-in signaling has successfully been studied, showing that TNF increases the number of binding sites on umbilical vein endothelial cells (HUVECs) and the adhesion of Jurkat cells to endothelium^22^.

In the study presented here, we employed SCFS and quantified the impact of TNF stimulation on lymphocyte adhesion in an endothelium-free, biomimetic fibronectin environment. The excellent force resolution of SCFS allowed us to study TNF-induced changes in lymphocyte adhesion both at the sub-cellular and the cellular level. Our results demonstrate that TNF enhances very initial cell-fibronectin contacts by reinforcing the forces associated with single rupture events and by increasing the number of bonds formed. This result provides quantitative evidence for the existence of an inside-out signaling mechanism involved in the TNF-mediated activation of lymphocyte adhesion.

## Results

### TNF increases cell detachment force and detachment energy on biomimetic fibronectin-coated surfaces

**Figure 1:**
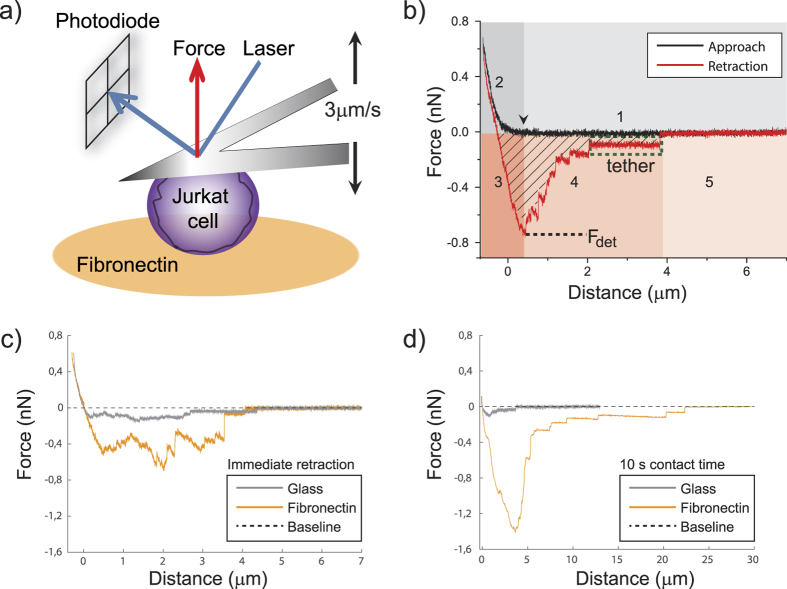
Adhesion of Jurkat cells quantified by Single-Cell Force Spectroscopy (SCFS). (**a**) Schematic of the force measurement setup. A single cell is attached to a tipless cantilever and brought into contact with a fibronectin-coated surface by an atomic force microscope (AFM). A laser beam is reflected off the back of the cantilever so that the position of the reflected laser beam at the photodiode is a measure of cantilever deflection and hence for the applied force. Cantilever approach and retraction movements were carried out at well-defined speeds (3 μm/s). (**b**) Representative force-distance curve for a single cell approached to the surface (black curve) and retracted again (red curve). The approach and retraction curves comprise the following phases: (1) the cell is not yet in contact with the surface. The first contact between cell and surface is marked with a black arrow; (2) the cell is deformed until reaching a pre-defined contact force; (3) after a pre-defined cell-surface contact time at maximum contact force the cantilever retracts while the cell is still adhering and is thus stretched (here: immediate retraction); (4) upon a certain force value (F_det_) cell detachment is initiated and individual, successive rupture events can be detected; (5) the cell is completely detached from the surface, hence force returns to zero. Membrane tethers (green box), as manifested by a negligible loading rate prior to a rupture event, are frequently observed during detachment (phase 4). The integral over the negative part of the retraction force curve (hatched area) represents the work of detachment. (**c**) Representative retraction curves for Jurkat cell adhesion on a fibronectin-coated versus an uncoated glass slide (control, immediate retraction). (**d**) Same as (**c**) for 10 s cell-surface contact time.

To explore whether TNF influences the adhesion of lymphocytes also in the absence of an *in vivo* endothelial cell layer, we carried out *in vitro* adhesion experiments on surfaces coated with fibronectin. Fibronectin is a ligand known to bind many different integrin types^23^ and it represents the natural coating of endothelial cell layers in blood vessels^24^. Jurkat cells were originally isolated almost 40 years ago from an Epstein Barr virus-negative, non-Hodgkin’s lymphoblastic leukemia^25^, followed by immortalization as a cell line, and, since then, have been widely used for studying signal transduction cascades and cell adhesion^26^,^27^,^28^,^29^. We ourselves have utilized Jurkat cells to study the TNF-FAN-RACK1-EED-nSMase2 signaling pathway^10^,^30^. Therefore, we considered them as a well-suited model system for studying signaling-related adhesion questions, such as the one addressed here. For quantifying the interaction of Jurkat cells with fibronectin as a function of TNF, we carried out SCFS experiments: a single living cell is attached to a tipless cantilever and pressed onto a fibronectin-functionalized surface using an atomic force microscope (Fig. 1a). After a defined time interval, the cell is detached from the surface with the cantilever and the force associated with the detachment of the cell from the surface is measured by determining the deflection of the cantilever. A representative force-distance curve (both approach and detachment) of a Jurkat cell on a fibronectin-coated surface is shown in Fig. 1b. We carried out experiments at three different cell-surface contact times, with the shortest one implying immediate retraction of the cantilever after reaching the maximum contact force. This corresponds to a time span of about 1.5 s between the first cell-surface contact and the initiation of cell detachment. For simplicity, we refer to this situation here as “immediate retraction”. The maximum contact force was set to 0.5 nN, but due to the intrinsic feedback of the AFM and drift correction it reaches between 0.5 and 0.9 nN. Figure 1c,d compare representative force-distance curves (retraction) for Jurkat adhesion on fibronectin-coated glass and bare glass, respectively, showing that fibronectin clearly strengthens Jurkat adhesion. We utilized serum-free medium to avoid any interference of growth factors and proteins that are present in serum and which might affect the adhesive properties of Jurkat cells in our experiments. In T cells, the α_4_β_1_ and α_5_β_1_ integrins are fibronectin-binding integrins and can modulate their activation^31^,^32^,^33^. In our Jurkat cells we found a much higher expression level of α_4_ compared to α_5_ integrins (Suppl. Fig. S1). Still, both integrin members contributed to Jurkat cell detachment forces (Suppl. Fig. S3).

**Figure 2:**
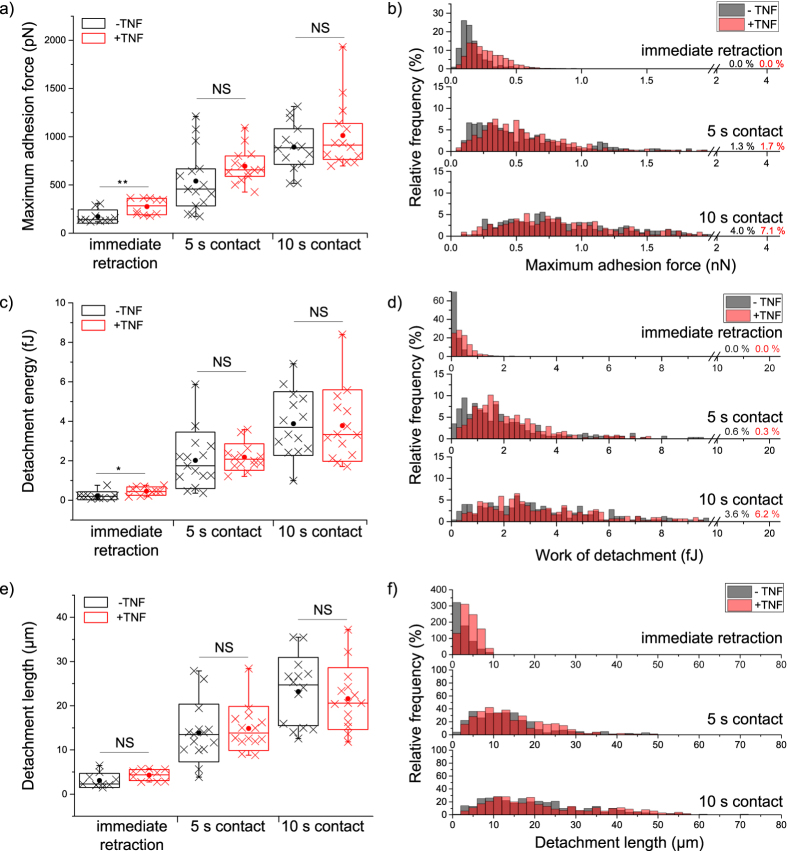
Influence of TNF on the adhesion of single Jurkat cells. Untreated (−TNF, black) and TNF-treated (+TNF, red) cells are compared and >11 cells were analyzed for each treatment and contact time situation. Every cross in (**a**,**c**,**e**) represents the average value of >15 force-distance curves from a single cell, i.e. an independent experiment (Box: interquartile range; line in each box: median; dot: mean; whiskers: minimum/maximum; box plots were also calculated on the basis of averaged values from independent cells). Relative frequencies in (**b**,**d**,**f**) were calculated from all recorded force-distance curves. Whereas **c**ell detachment forces (**a**,**b**) and work of detachment (**c**,**d**) are significantly influenced by TNF at the shortest contact time, the detachment length is not affected by TNF. Statistical significance was tested by applying a Student’s t-test to cell averages, i.e. independent experiments (**p < 0.01, *p < 0.05, NS: no significant difference at p < 0.05).

For a quantitative comparison of Jurkat cell adhesion as a function of TNF stimulation, we determined the force and work required to detach Jurkat cells from fibronectin-coated glass. Different cell-surface contact times (immediate retraction, 5 s, 10 s) were evaluated in order to learn about time-dependent effects of TNF stimulation. As shown in Fig. 2, both cell detachment forces and the work of detachment increase with increasing cell-surface contact time. Most importantly, in response to TNF-stimulation, the cell detachment force increases in average by 61% compared to non-stimulated cells (Fig. 2a,b; significance level <0.01, Student’s t-test) for the shortest contact time. However, TNF does not significantly influence cell detachment force at longer contact times. A similar effect of TNF stimulation has been observed for the work of detachment (Fig. 2c,d). For the shortest contact time, the work of detachment is approximately doubled (105% increase) for cells stimulated with TNF, whereas for 10 s contact time no such increase can be detected at all. Interestingly, the detachment length (i.e. the position of the last rupture event in the force-distance curve) is not influenced by TNF (Fig. 2e,f). The dispersion of measured values arises on the one hand from cell-to-cell variations, and on the other hand it is an intrinsic property of cell adhesion kinetics and the statistical nature of cellular binding and unbinding processes due to the relatively low binding energies of biological bonds^34^,^35^.

### TNF increases the number of single rupture events in a force curve

**Figure 3:**
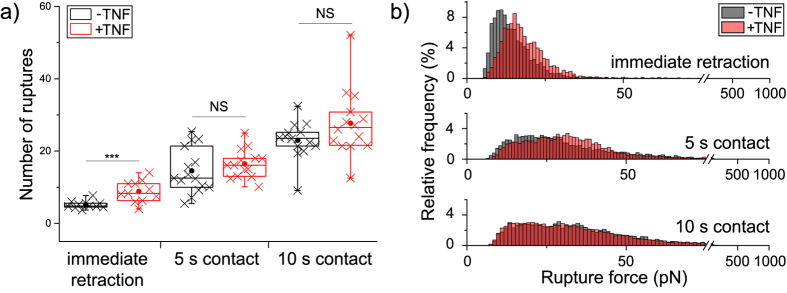
Parameters derived from single rupture events in force-distance curves. (**a**) Number of rupture events per curve. Each cross represents the average value measured for one cell, and for each cell >15 force-distance curves were analyzed. Statistical significance was tested at the level of cell averages by a Student’s t-test (***p < 0.001, NS: no significant difference at p < 0.05). If the cell is immediately retracted after reaching full contact, TNF causes a clear increase in the number of rupture events. **(b)** Relative frequency of forces associated with single rupture events detected during cell detachment. For immediate cell retraction, >2000 rupture events were analyzed. At 5s and 10 s contact time, >5000 rupture events were analyzed and a clear shift towards larger forces is evident for the shortest contact time in response to TNF stimulation. With increasing contact time, the TNF-induced reinforcement of ruptures disappears.

To further investigate if the TNF-induced reinforcement of Jurkat adhesion observed for the cell detachment forces can be explained by changes in molecular binding processes, we carried out a detailed analysis of single ruptures in force-distance curves as a function of TNF stimulation (phase 4 in Fig. 1b). An increase in the number of bonds present at the end of the adhesion phase could be a first parameter explaining the TNF-induced increase in cell detachment force. Indeed, TNF stimulation significantly increases the average number of rupture events needed to completely detach a cell from the surface for the shortest contact time (Fig. 3a). At longer contact times this TNF-induced increase disappears, in agreement with the results obtained for the total cell detachment forces.

### TNF-stimulation increases the force associated with single rupture events

To investigate whether the TNF-induced increase in cell detachment force is, in addition to the increased number of rupture events, additionally caused by a reinforcement of single rupture events, we analyzed the forces associated with single rupture events in force-distance curves. Figure 3b shows the relative frequency of forces associated with single rupture events in cells as a function of TNF treatment. Interestingly, stimulating cells with TNF clearly shifted the relative frequencies of single rupture forces and the most frequent rupture force towards higher forces at the shortest contact time (Fig. 3b). For a contact time of 5 s, still a tiny shift of relative frequencies towards higher forces is evident, whereas for 10 s contact time no reinforcement of single rupture events can be observed.

### Final rupture forces before cell detachment are influenced by TNF

**Figure 4:**
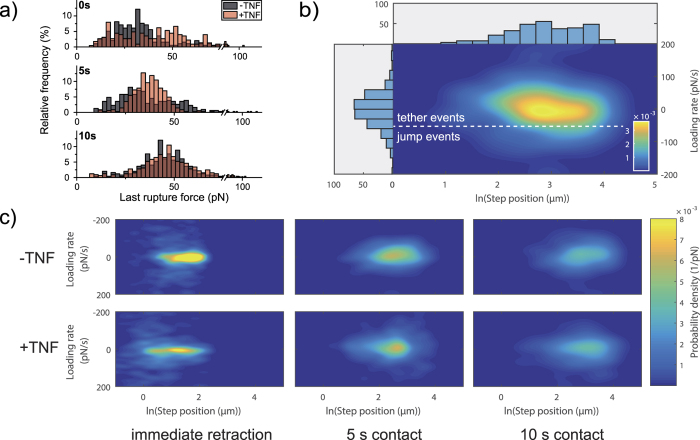
Analysis of the final rupture events in a detachment curve. (**a**) Force associated with the last rupture event before complete cell detachment. At least 200 curves were analyzed for each experimental situation. (**b**) Principle of analyzing the cytoskeletal coupling using a probability distribution heatmap. Dashed line: Estimation of the threshold between tether events (membrane anchorage) and jump events (cytoskeletal anchorage). **(c)** Probability density distributions for the loading rate of the last rupture step and the corresponding step position clearly show that bonds are mainly anchored by tethers. More than 15 force-distance curves per cell from at least 11 cells were taken into account for generating each heatmap.

With SCFS, it is neither possible to determine the order in which molecules detach nor to measure how the load is exactly distributed among the bonds during the detachment phase. Probably, adhesion sites at the rim of the adhesion zone rupture first, but due to the complex force and bond distributions in the cell-surface contact zone, it is also possible that the molecules just rupture in a very random way^35^. Therefore, the last rupture, directly before complete cell detachment takes on a special role, as for this very last rupture, influences from other cell-surface connections can be excluded. Hence, analyzing the force associated with the last rupture event can give a variety of information on the properties of single interactions between cell and surface. Consistent with the result shown in Fig. 3b, we find for the final rupture events that TNF broadens the relative frequencies of forces, such that more events are recorded at higher force values (Fig. 4a) for the situation of immediate cell retraction. Furthermore, it is striking that forces associated with the last rupture event have a very broad distribution at the shortest contact time, whereas their distribution appears less spread for 10 s contact time and the majority of rupture events occurs at higher forces compared to the situation of immediate cell retraction. It is also noteworthy that forces associated with the last rupture event are in average higher compared to the forces shown in Fig. 3b. This difference can result from the compensation of the rupture-induced change in cantilever deflection by many still active connections with the surface, so that a reduced cantilever deflection change is measured. The last rupture is indeed the decisive event for recording single molecule forces, as all intermediate ruptures are only low-end estimates and can be biased by the active connections between cell and surface^19^.

A particularly interesting parameter is the loading rate, which reflects the mechanical properties of the single bond anchorage prior to bond rupture. One can here distinguish between so-called tether events and jump events^36^. In tether events the bond is viscously anchored, presumably through a membrane tube; in jump events, an elastic coupling is provided and significant loading is applied to the bond and the measured rupture increases with increasing loading rate^37^,^38^. Recently, Sariisiik *et al*. have reported a novel analysis method to distinguish between tether and jump events in SCFS data by plotting the probability distribution of the slope prior to a rupture event against the position of the rupture event (Fig. 4b)^39^. As shown in Fig. 4c, in our experiments the final rupture event is clearly dominated by tethers, regardless of TNF stimulation. Hence, TNF does not increase the type of cytoskeletal coupling in the Jurkat-fibronectin bonds so that their intracellular mechanical anchorage is not changed by TNF. Whereas for immediate cell retraction a very narrow distribution of slopes is observed, the distribution broadens with increasing contact time. We attribute this broadening to the natural change in the mechanical anchorage of bonds over contact time.

## Discussion

We have used single-cell force spectroscopy (SCFS) for proving the existence of a long-hypothesized TNF-induced inside-out-signaling mechanism that reinforces integrin-mediated Jurkat cell adhesion. In order to separate effects caused by inside-out signaling from those induced by outside-in signaling, we investigated the forces required to detach single cells from fibronectin-coated surfaces in an endothelium-free *in vitro* environment. With our SCFS-based method, we quantified cell adhesion as a function of TNF-stimulation at the cellular level and at the level of single rupture events. Our results show that after TNF stimulation, Jurkat cells display a remarkable increase of both cell detachment force and work of detachment, but only at the shortest contact time (Fig. 2). This result is in agreement with the requirements of the particular biological situation, where TNF should activate integrin-mediated adhesion on fibronectin (i.e. integrin α_L_β_2_, integrin α_4_β_1_)^27^ in order to arrest T cells prior to invading the surrounding tissue. Stopping the initially rolling lymphocytes must therefore be fast and efficient, which can be achieved by strong adhesion at short interaction times. In a previous study focusing on the adhesion of T-lymphocytes to TNF-activated endothelial cells (HUVECs, 6 h of TNF treatment; outside-in signaling), the pre-upregulated integrin ligands ICAM-1 and VCAM-1 contributed to firm adhesion to integrins in a very fast manner (0.25 second) which is similar to the timeframe that we observe here^22^. Furthermore, experiments on leukocyte adhesion under laminar flow conditions have shown that sub-second timescales are indeed relevant for the cytokine-induced activation of α_4_ integrin-mediated adhesion and for integrin α_4_ clustering^40^.

By further analyzing the force-distance curves in detail, we found two mechanisms that are presumably the main reasons for the observed TNF-induced reinforcement of initial cell adhesion, i.e. at the shortest contact times: Then, (i) the number of rupture events required to completely detach a cell from the surface is increased in TNF-stimulated cells, and (ii) the forces associated with single rupture events are reinforced by TNF-stimulation (Fig. 3). Note, that the contact time where an effect of TNF stimulation has been observed (i.e. about 1.5 s) is by far too short for *de novo* protein expression to occur (any effect on protein expression caused by the 25 min TNF treatment will be detected as background in the measurements) and there is, to the best of our knowledge, no study reporting an enhanced level of α_4_β_1_ expression due to TNF stimulation. Therefore we attribute the effects that we have observed to the stabilization of single rupture events, which is presumably to a significant extent due to a switching of integrins from an inactive to active state, i.e. TNF induces an increase of binding affinity. An additional effect might be that binding avidity, i.e. the number of binding competent molecules that are available in the membrane to be accessed by the binding partner^17^, are increased. Future studies using smaller contact forces^41^ and even shorter contact times should be able to distinguish between both effects, i.e. the influence of TNF-stimulation on binding affinity and binding avidity.

Interestingly, we have determined a large increase of the forces associated with the last rupture events with increasing cell-surface contact time (Fig. 4a). TNF has indeed been reported to facilitate cluster formation at very short contact times or to lead to the formation of clusters in lipid rafts^42^,^43^,^44^, so then no single bonds but instead clusters of integrins would bind preferably. Such a cluster formation of α_4_ integrins has recently also been reported for leukocyte adhesion under shear stress^40^, and clustering at short timescales is also well-known for other types of integrins^45^. Hence, the increase in single rupture force could indicate the involvement of molecular clusters in our experiments. Such an integrin clustering could also explain the broad force distribution at the shortest contact time, where forces in the range from about 10 to 100 pN have been measured, particularly for TNF-stimulated cells. At 10 s contact time the force distributions have almost Gaussian appearance around a mean value and TNF-stimulation then clearly does not influence rupture forces. This indicates that integrin binding to fibronectin then (i.e. at longer timescales) itself induces integrin cluster formation, similar to the clustering of α_v_β_3_ integrins in focal adhesions^46^.

In all our experiments, we consistently observe that cell detachment forces increase with contact time, while at the same time the effect of TNF-induced adhesion reinforcement becomes less relevant with increasing contact. This increase in cell detachment force with contact time is a typical result and can on the one hand be due to an increase in the number of bonds until a constant cell adhesion area, and hence binding equilibrium, is reached. On the other hand, it can be due to a stabilization of the ligand-receptor complex^47^,^48^. Taken together, this could mean that TNF-stimulated cells are more adhesive in the beginning of their adhesion period, but the interaction with fibronectin additionally activates integrins independently of TNF, resulting in almost equal adhesion of +/−TNF cells after 10 s.

An important strategy to investigate if the mechanical coupling of molecular bonds involved in cell adhesion is influenced by certain experimental conditions is the comparison of the cell detachment forces and the work of detachment^49^. Our results show a similar TNF-induced increase of both the cell detachment force and the work of detachment (Fig. 2), indicating that TNF does not significantly influence the energy dissipated during the cell detachment process, as it would be the case if the cell body became more viscous. Additionally, distinguishing between tether events and jump events can give even further important information on the cytoskeletal coupling of single biomolecules^36^,^39^,^50^. We clearly observed that single rupture events are dominated by tethers and that their cytoskeletal coupling is not influenced by TNF-stimulation (Fig. 4). This is a very interesting aspect, as in monocytes high affinity α4β1/VCAM-1 complexes have been reported to be rather linked via membrane tethers than via cytoskeletal anchorages^51^.

As an important response to extracellular stimuli, Bouaouina and co-workers found that the phosphorylation of p38 in floating neutrophils can be detected after 10 min with TNF incubation, and the level was reduced after 60 min^6^. Similarly, protein kinase C has been reported to activate the α_2_β_1_ mediated inside-out signaling pathway within a short amount of time^52^. Cells in our experiments were incubated with TNF for 25 minutes prior to starting the experiments. TNF-induced activation of nSMase2 needs 90 seconds to reach its maximum^10^, so that in our experiments all the necessary signaling pathways should have been activated.

**Figure 5:**
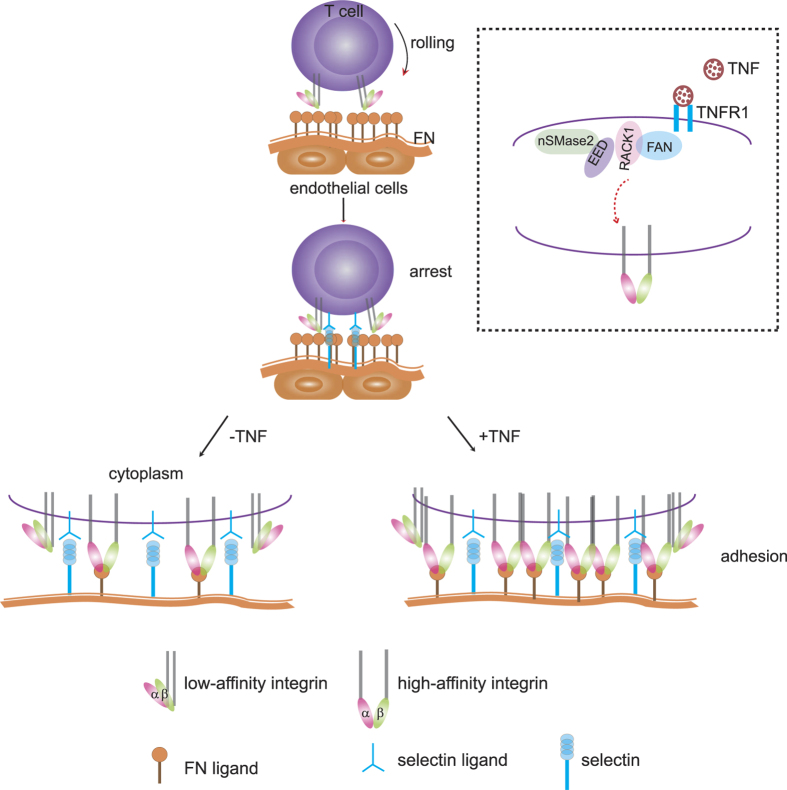
Model for T cell adhesion to the endothelium in the presence (+TNF) and absence of TNF (−TNF) *in vivo*. Fibronectin covers the endothelial cell layer. When T cells are rolling and arrested, integrins on the membrane of T cells are in a low affinity conformation. Arrest occurs mainly via selectins on the endothelial cells and their respective ligands on T cells. When cells are stimulated with TNF, a higher proportion of activated integrins is recruited to the adhesion site and integrin clusters are formed in comparison to the situation without TNF stimulation. More bonds are present after TNF stimulation. Inset: Hypothetical model for activation of inside-out signaling by TNF. As previously shown^10^, TNF stimulation induces the formation of the FAN·RACK1·EED·nSMase2 complex. Due to the reported ability of RACK1 and EED to interact with integrins^12^,^13^,^14^, we propose that this complex may directly bind to the integrins (shown as heterodimer) and thus transduces force to the extracellular space.

Figure 5 summarizes our current view of TNF-induced lymphocyte adhesion reinforcement *in vivo* by a combined effect of inside-out and outside-in signaling. Integrins are directly activated by TNF, their binding to fibronectin is facilitated and bonds are reinforced, leading in turn to an increase in the number of bonds between the lymphocyte and the fibronectin layer situated on top of the endothelial cells. As shown in the inset of Fig. 5, we have recently described a signaling pathway of TNF which involves a complex of TNF-R1 (the 55 kDa receptor for TNF), the proteins FAN, RACK1, EED and the lipase nSMase2^10^. Remarkably, both RACK1 and EED have been described as integrin-interacting proteins^12^,^13^,^14^ and nSMase2 has been linked to (integrin-mediated?) contact inhibition^10^. Based on these published data, we hypothesize that TNF may trigger integrins via this pathway (Fig. 5, Inset, lower part) and intend to clarify this in future studies. Taken together, our data strongly support the notion that T-lymphocyte adhesion is enhanced by a TNF-induced inside-out signaling, which acts on integrins.

The observed very strong effect of TNF on initial cell adhesion events suggests that TNF rather strengthens short-term binding events, such as single molecules and possibly the formation of initial adhesion clusters, e.g. in lipid rafts. Integrin transport in lipid rafts has previously been shown for a different integrin type in T lymphocytes^44^. In summary, this work provides for the first time a direct demonstration of the presence and relevance of an inside-out signaling process for the reinforcement of integrin-mediated adhesion of T-lymphocytes in an endothelium-free, biomimetic environment. Notably, our current study has been performed using Jurkat cells as a model for T lymphocytes. These cells have been used to analyze T cell biology for many years and in many respects, but it is also clear that they differ from primary T cells in many ways, e.g. by lacking the phosphatases PTEN and SHIP^28^. Similarly, Riley and coworkers found many similarities but also many differences in the expression profiles of Jurkat vs. primary T cells after stimulation of the TCR and costimulatory receptors^53^. With regard to adhesion, resting, unstimulated primary T cells reportedly do not show significant binding to integrin ligands whereas certain Jurkat cell lines already show a high ligand-binding state, at least for the integrin ligand VCAM-1^54^. Therefore it will be of interest to determine whether primary T cells show even more pronounced effects after TNF stimulation in future experiments. Likewise, further studies will be needed to determine the detailed biochemical signaling involved in Jurkat, but also in primary T cells or in T cell lines.

In conclusion, we provide clear evidence for the reinforcement of lymphocyte adhesion by TNF inside-out signaling, thereby highlighting a novel function of TNF in the regulation of immunological adhesion of T lymphocytes and pointing to yet another, so far undiscovered, role of TNF in T cell-mediated adaptive immunity.

## Methods

### Cell culture

Jurkat E6-1 cells were originally obtained from the ATCC and cultured in Click’s/ RPMI 1640 medium (Applichem, Germany) containing 10% v/v fetal bovine serum (Biochrom, Germany), 100 μg/ml penicillin and streptomycin (Biochrom) at 37 °C, 5% w/v CO_2_, and about 90% humidity. Cells were passaged twice a week and were maintained up to passage 10. For stimulation with TNF, cells were incubated with TNF (100 ng/ml) (BASF Bioresearch, Germany) in culture medium at 37 °C for 25 min prior to an experiment.

### Fibronectin functionalization

For preparing the biomimetic adhesion environment, a coverslip was cleaned with 70% v/v ethanol and glued into a Petri dish (TPP, Switzerland) with biocompatible glue (JPK Instruments, Germany). Human fibronectin (Sigma-Aldrich, Germany) was physisorbed on the coverslip at 4 °C overnight at a concentration of 15 μg/cm^2^. To remove excess proteins, the fibronectin surface was washed several times with 1x PBS.

### Single-Cell Force Spectroscopy (SCFS)

Cell adhesion forces were measured using atomic force microscopy (AFM). In detail, a NanoWizard 3 Bioscience with piezo range up to 15 μm (JPK Instruments, Germany) was used for the shortest cell-surface contact time and a CellHesion 200 (JPK Instruments, Germany) with 100 μm piezo travel length was used for longer cell-surface contact times (5 s, 10 s) in order to make sure that the cell detachment length can fully be reached with the piezo. Tipless cantilevers (MLCT-O10, Bruker, Germany) were rinsed once with acetone before calibrating their spring constants in a fluid chamber (BioCell, JPK Instruments, Germany) filled with 1 × PBS. Cantilevers B, C, and D with nominal spring constants between 10 and 30 mN/m were chosen. Calibration was carried out at room temperature according to the thermal noise method with correction for hydrodynamic effects using standard routines implemented in the JPK AFM software, leading to averaged calibrated spring constants from 14 to 46 mN/m. Calibrated cantilevers were incubated in biotin-BSA (0.5 mg/ml in 1 × PBS, Sigma-Aldrich, Germany) overnight at 37 °C in a Petri dish wrapped with parafilm. Afterwards, they were incubated with streptavidin (0.5 mg/ml in 1 × PBS; S4762, Sigma-Aldrich, Germany) for 10 minutes at room temperature, and then incubated 10 minutes in biotin-concanavalin A (0.2 mg/ml in 1 × PBS, Sigma-Aldrich) at room temperature^21^. Between each functionalization step, cantilevers were rinsed extensively with 1 × PBS. Concanavalin A was used to coat the cantilevers, as Jurkat cells bind to this lectin and it has successfully been used in SCFS experiments on Jurkat cells by others^22^,^55^.

For the adhesion force experiments, 2 ml serum-free RPMI 1640 medium (Gibco, Germany) supplemented with 100 μg/ml penicillin/streptomycin (Biochrom, Germany) were added into the Petri dish containing the fibronectin surface. All experiments were carried out at approximately 36 °C in a heated fluid chamber (PetriDishHeater, JPK, Germany). The functionalized cantilever was approached to the surface with the AFM and cantilever sensitivity was measured. For attaching a living cell to the free end of the cantilever, a droplet of cell suspension was pipetted into TNF-free medium in the Petri dish far away from the fibronectin-coated coverslip. Then (lag time ≈ 20 s), the cantilever was pressed to a cell for several tens of seconds to guarantee successful attachment. After cell attachment, the cantilever was lifted upwards and moved above the fibronectin-coated coverslip. The cell was allowed to relax at the cantilever for about 2 minutes before cell adhesion forces were measured for the first time. This procedure was exactly the same for all experimental situations. In the cell adhesion force experiments, the cell was approached to the surface at a speed of 3 μm/s until a maximum contact force of 500 pN was reached before it was retracted at a speed of 3 μm/s. During cantilever retraction the deflection of the cantilever was recorded. All experiments were carried out in closed-loop and constant height mode, i.e. the cantilever position was kept at a constant height after reaching the maximum contact force. We optically confirmed (IX71, Olympus) that the cell did not change its position during the experiment in order to exclude data from loosely attached cells. 11–15 cells were used for experiments in each experimental situation (i.e. for each contact time and with/without TNF treatment) and for each cell at least 15 force-distance curves were recorded. Hence, the maximum timespan between adding the cells to the liquid chamber until finishing the experiment was about 5–10 min. Suppl. Fig. S4 shows the results from control experiments on bare glass.

### Data analysis

Force-distance curves were analyzed with a commercial data processing software (JPK Data Processing Version spm-5.0.78, JPK Instruments, Germany), where the detection of single rupture events is based on a previously published algorithm^56^. Results were plotted with Origin 9.0 (Originlab, USA). Statistical analysis was carried out with a Student’s t-test in Origin 9.0 (Originlab, USA).

The probability density distributions of the logarithmic rupture position and the slope (Fig. 4) were calculated using a bivariate gaussian kernel density estimator^57^ in MATLAB (The MathWorks, Natick, MA) and were plotted as heatmaps (also in MATLAB). For the loading rate analysis the interval between 200 pN/s and −200 pN/s was chosen.

## Additional Information

**How to cite this article**: Li, Q. *et al*. Reinforcement of integrin-mediated T-Lymphocyte adhesion by TNF-induced Inside-out Signaling. *Sci. Rep.* **6**, 30452; doi: 10.1038/srep30452 (2016).

## Supplementary Material

Supplementary Information
