# Supplementary material for: Reinforcement of integrin-mediated T-Lymphocyte adhesion by TNF-induced Inside-out Signaling
Source: Sci Rep. 2016 Jul 28;6:30452. doi: 10.1038/srep30452 (PMC4964354; doi:10.1038/srep30452)
Supplement: Supplementary Information [file srep30452-s1.pdf]

Supplementary information for:

Reinforcement of integrin-mediated T-Lymphocyte adhesion by TNF-induced Inside-out Signalling

Qian Li, Steven Huth, Dieter Adam, Christine Selhuber-Unkel

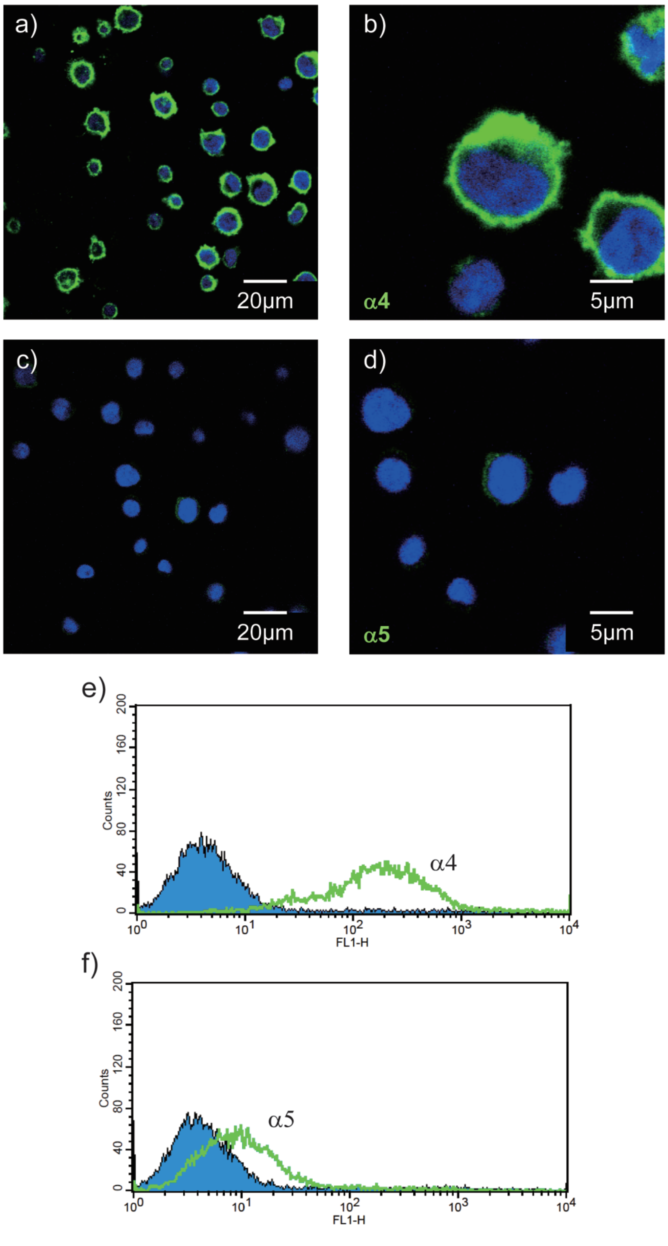

Supplementary Figure S1| Integrin expression in Jurkat E6-1 cells investigated with confocal laser scanning microscopy (a-d) and flow cytometry (e, f). (a, b) Expression of integrin α_4 or (c, d) integrin α_5 (green) was investigated in Jurkat E6-1 T lymphocytes. Nuclei were stained with DAPI (blue). In (e and f), the green lines depict α_4 and α_5-positive cells, blue peaks the corresponding isotype controls. For immunofluorescence, 10^6 cells were seeded on poly-L-lysine-coated (100 μg/ml) coverslips (30 min, 37 °C), fixed with 4% w/v PFA (Sigma-Aldrich, 25 min room temperature), washed twice with PBS, permeabilized with PBS/1% v/v Triton X-100 (Sigma-Aldrich, 5 min) and blocked with PBS/1% w/v BSA (Sigma-Aldrich, 1h). Cells were incubated with monoclonal anti-human CD49d/α_4 or CD49e/α_5 antibodies (ImmunoTools, Germany, 10 μg/ml in 1% w/v BSA overnight, 4 °C), washed 3 x with PBS, incubated with FITC-conjugated secondary antibody (goat anti-mouse IgG, (H+L), Millipore, Germany, 10 μg/ml in 1% w/v BSA, 37 °C, 1 h), extensively washed with PBS and analyzed with a confocal laser scanning microscope (Carl Zeiss Jena, Germany). For flow cytometry, 2.5×10^5

cells were collected at 4 °C, washed twice with PBS/1% w/v BSA, stained with the above primary (4 °C, 1 h) and secondary antibodies, washed again twice and fixed with 1% w/v PFA. Isotype controls were stained with mouse IgG1 or IgG2a (BD, Germany). Green fluorescence was measured on a FACSCalibur flow cytometer using the BD CellQuest^{TM} Pro software V.4.0.2.

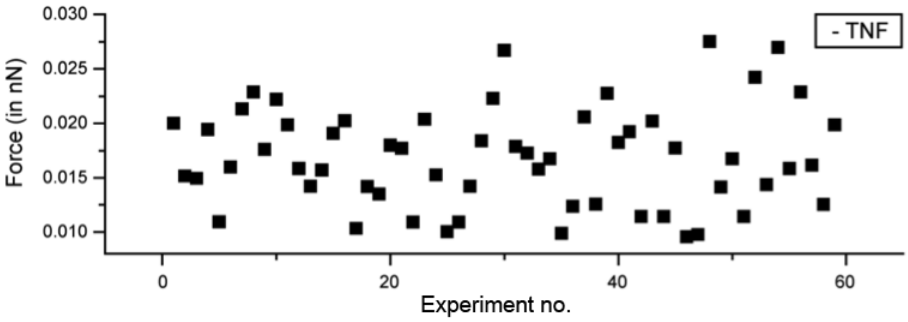

Supplementary Figure S2| Distribution of last rupture forces of a single cell over the course of an experiment (60 force-distance curves). No drift or significant change has been observed. The shortest cell-surface contact time was used (i.e. immediate retraction of the cantilever after reaching the maximum contact force) and the cell was not treated with TNF.

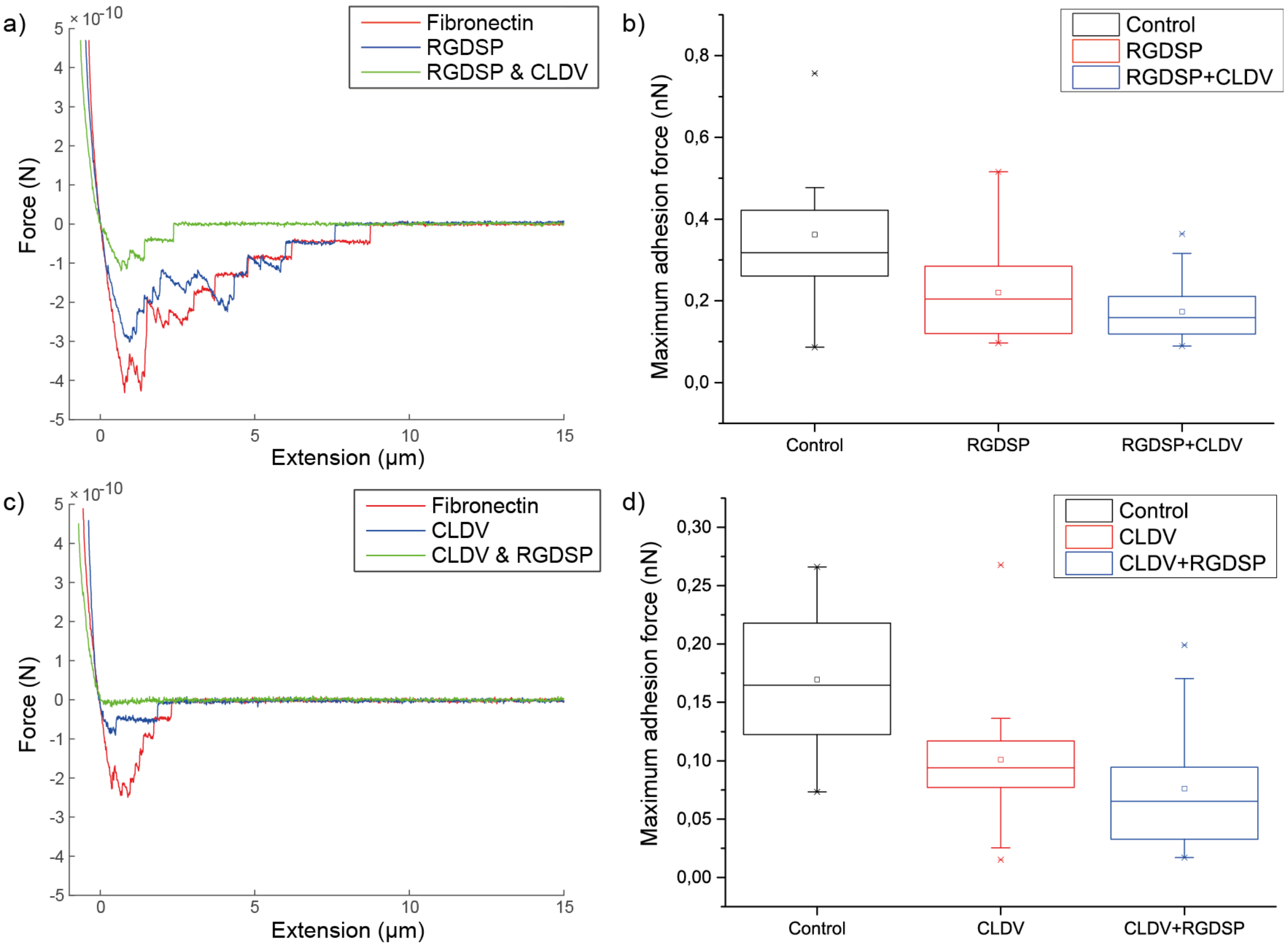

Supplementary Figure S3|Adhesion experiments in the presence of integrin blocking peptide. Cantilever functionalization, substrate preparation and cell fishing were realized as described in the methods section. After cell attachment, 20 force curves were taken with a setpoint of 500 pN and a velocity of 3 µm/s in constant height mode (immediate retraction) on the fibronectin surface without any blocking agents (control). The first blocking peptide was added to a concentration of 200 µM using the inlets of the petridish heater

(*in situ* using one and the same cell to avoid cell-to-cell deviations*)*. After an incubation time of 40 minutes, 20 force curves were taken with the same cell. Then, the second blocking agent was added to a concentration of 200 µM and another 20 force curves were recorded. The experiment was repeated adding first the RGD and then the LDV. a) and b) show force-extension curves and results for the control, then adding RGD (GRGDSP, Sigma, Germany; binds to $\alpha_5\beta_1$ integrins) and then LDV (WLDVC, Biosyntan, Germany; binds to $\alpha_4\beta_1$ integrins). In c) and d) the order of LDV and RGD addition was exchanged. In (b), RGD addition caused in average a force reduction of 39%, LDV led to a further reduction of about 21%. In (d), LDV addition reduced the average force by about 40%, subsequent RGD addition by about 25%. Hence, both $\alpha_4\beta_1$ and $\alpha_5\beta_1$ integrins significantly contribute to cell detachment forces.

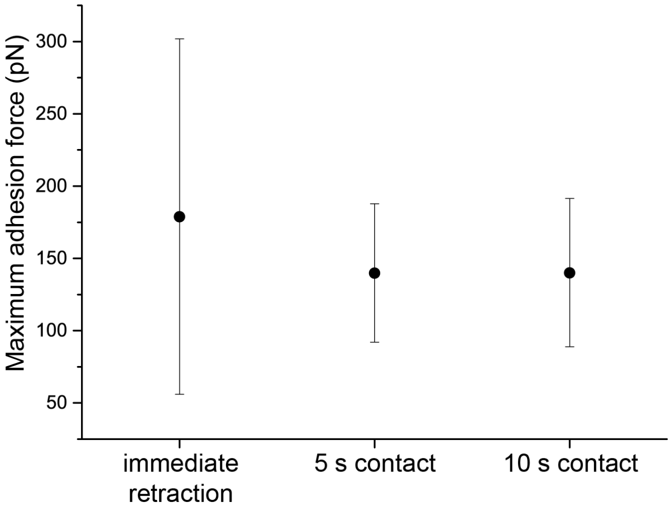

Supplementary Figure S4| Mean cell detachment forces (error bars: standard deviation) on unfunctionalized glass as a function of contact time. These forces are clearly smaller than the forces measured with fibronectin on the glass (cf. figure 1 in main manuscript). For the case of immediate retraction, data from 11 cells, each 6-28 force-extension curves (in total 158 force-extension curves), were taken into account. For 5 s contact time, 41 curves from 2 cells were used for calculating the average values (20-21 curves per cell). For 10 s contact time, 38 force-extension curves from 2 cells (18-20 curves per cell) were averaged. Error bars denote standard deviation.
